# Synthetic-to-real: instance segmentation of clinical cluster cells with unlabeled synthetic training

**DOI:** 10.1093/bioinformatics/btac219

**Published:** 2022-06-27

**Authors:** Meng Zhao, Siyu Wang, Fan Shi, Chen Jia, Xuguo Sun, Shengyong Chen

**Affiliations:** Engineering Research Center of Learning-Based Intelligent System (Ministry of Education), The Key Laboratory of Computer Vision and System (Ministry of Education), and the School of Computer Science and Engineering, Tianjin University of Technology, Tianjin 300384, China; Engineering Research Center of Learning-Based Intelligent System (Ministry of Education), The Key Laboratory of Computer Vision and System (Ministry of Education), and the School of Computer Science and Engineering, Tianjin University of Technology, Tianjin 300384, China; Engineering Research Center of Learning-Based Intelligent System (Ministry of Education), The Key Laboratory of Computer Vision and System (Ministry of Education), and the School of Computer Science and Engineering, Tianjin University of Technology, Tianjin 300384, China; Engineering Research Center of Learning-Based Intelligent System (Ministry of Education), The Key Laboratory of Computer Vision and System (Ministry of Education), and the School of Computer Science and Engineering, Tianjin University of Technology, Tianjin 300384, China; School of Medical Laboratory, Tianjin Medical University, Tianjin 300204, China; Engineering Research Center of Learning-Based Intelligent System (Ministry of Education), The Key Laboratory of Computer Vision and System (Ministry of Education), and the School of Computer Science and Engineering, Tianjin University of Technology, Tianjin 300384, China

## Abstract

**Motivation:**

The presence of tumor cell clusters in pleural effusion may be a signal of cancer metastasis. The instance segmentation of single cell from cell clusters plays a pivotal role in cluster cell analysis. However, current cell segmentation methods perform poorly for cluster cells due to the overlapping/touching characters of clusters, multiple instance properties of cells, and the poor generalization ability of the models.

**Results:**

In this article, we propose a contour constraint instance segmentation framework (CC framework) for cluster cells based on a cluster cell combination enhancement module. The framework can accurately locate each instance from cluster cells and realize high-precision contour segmentation under a few samples. Specifically, we propose the contour attention constraint module to alleviate over- and under-segmentation among individual cell-instance boundaries. In addition, to evaluate the framework, we construct a pleural effusion cluster cell dataset including 197 high-quality samples. The quantitative results show that the numeric result of *AP*^mask^ is > 90%, a more than 10% increase compared with state-of-the-art semantic segmentation algorithms. From the qualitative results, we can observe that our method rarely has segmentation errors.

## 1 Introduction

The comprehensive segmentation of cells are core analysis steps in many histopathology image analysis tasks (Bazgir *et al.*, 2021; Gurcan *et al.*, 2017; Saltz *et al.*, 2017; Shang *et al.*, 2021). A number of studies have been performed in the segmentation of cells. Gençtav *et al.* (2012) and Song *et al.* (2017) propose a framework based on prior knowledge and automatic threshold to gradually separate cervical cell clump from the background, cell nuclei and cytoplasm boundary from the cervical cell regions. Unet (Yang *et al.*, 2017) and its variants (Isensee *et al.*, 2021; Zhao *et al.*, 2019b) use skip connection to integrate high-level semantic and low-level fine-grained texture information to improve epithelial nucleus segmentation quality (Yi *et al.* 2019a; Zhou *et al.*, 2019b) joint detector (SSD, Fastercnn) and segmenter (FCN, Unet) to achieve neural cell and cervical cell instance segmentation.

**Fig. 1.**
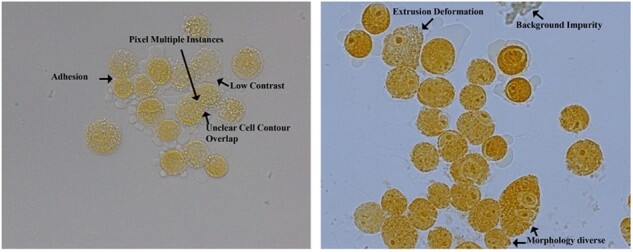
Illustration of morphology pleural effusion cluster cells. Pleural fluid tumor cluster cells often have variant shapes, overlap, adhesion, obscure contour, low contrast, deformation, background impurity, which is very easy to lead to false detection, missed detection, over- and under-segmentation

However, the above methods perform poorly in tumor cluster cells, such as Pleural effusion tumor cluster cells (Sarioglu *et al.*, 2015; Win *et al.*, 2017) illustrated in Figure 1. There are three main reasons for these problems: (i) Model generalization. Due to the cluster cell morphology being varied and the segmentation methods (Gençtav *et al.*, 2012; Song *et al.*, 2017) rely heavily on cells’ prior knowledge. Therefore, these methods only using fixed prior knowledge perform poorly in new miscellaneous cells segmentation tasks. (ii) Multiple instances properties. The cell pixels in the overlapping or adhesion area belong to multiple instances. Some semantic segmentation methods such as Isensee *et al.* (2021), Yang *et al.* (2017) and Zhao *et al.* (2019b) can only define that cell pixels in the overlapping the area belongs to one instance, which often lead to error distinguishment of cell pixels. (iii) Cluster properties. Due to the distortion, adhesion, overlap between cells, the cell contour is blurred and the contrast is low. It is not sufficient for Yi *et al.* (2019a) and Zhou *et al.* (2019b) to use mask only to regress the cell obscure boundary. Therefore, these methods often lead to over-, under-segmentation, false and missed detection of cell pixels. In addition, labeling cluster cell with blurred outlines requires the professional guidance of several pathologists, which is a time-consuming and laborious process.

Overall, how to high-quality segment cluster cells is still a significant challenge. Therefore, we propose a contour constraint instance segmentation framework (CC framework) without prior knowledge based on cluster cell combination enhancement (CCCE). First, to alleviate the demand for a large amount of data in the network framework, we constructed a data enhancement module CCCE, which can enrich cluster cell information through the small number of discrete cells. Secondly, to avoid false detection and missed detection in intertwined complex cell regions, inspired by keypoints detection (KD; Yi *et al.*, 2019b), detect branches of CC framework (KD module) outputs top-left, top-right, bottom-left, bottom-right and the center points of a cell on multiple scales. Then, each cell rectangle can be generated by three points or any two diagonal points in five points, which can distinguish multiple instances of a cell of pixel and further improve the detection accuracy and avoid missed detection. Finally, in the cell segmentation branch, to prevent over- or under-segmentation in the fuzzy region of ROI multi-instance cells, we fuse the deep to the shallow features to recover the boundary information and construct the contour attention constraint (CAC) module to constrain the cell boundaries. The effect of constraint boundary can also further enhance the expression ability of key points when network parameters are updated by backpropagation.

In summary, our main contributions are given as follows.

1. We construct a pleural effusion cluster cell dataset including three categories: Discrete pleural effusion cells (68 labeled images), which are used to synthesize aggregated pleural effusion cells and labels; Synthetic pleural effusion cluster cells, which consist of 200 samples as the training set in our framework; Real pleural effusion cluster cells, which consist of 129 labeled images as the testing set in our framework. This dataset provides great challenges in medical image segmentation, such as overlapping, touching, low contrast and complex background.
2. We propose a cluster cell instance segmentation framework that only requires unlabeled synthetic dataset as the training set and achieves an high-quality cell boundaries instance segmentation close to fully supervised methods on real pleural effusion tumor cluster cell dataset. The framework includes three modules: CCCE, KD and contour attention constraint segmentation (CACS). The CCCE module can simulate the clusters cells image by using only a few discrete cells images, which greatly reduces the dependence of the network on the dataset. The KD and CACS module can be applied to various medical segmentation images, especially the cluster cell images of complex scenes.
3. We compare with five state-of-the-art (SOTA) algorithms on two datasets. Our algorithm achieves a 13% increase compared with other segmentation algorithms and attains >97% on metric *AP*^mask^ (averaged over Mask IoU thresholds). In metric $AP_{\text{TP}}^{\text{boundary}}$ (averaged over Boundary IoU thresholds), the framework also achieves comparable results.

### 1.1 Related work

#### 1.1.1 Image data enhancement

Data augmentation enhances the size and quality of training datasets that are widely used in deep learning networks. For example, some scholars have proposed regularization methods [Dropout (Srivastava *et al.*, 2014), Cutout (DeVries and Taylor, 2017), Mixup (Zhang *et al.*, 2017) and Gridmask (Chen *et al.*, 2020a)] to solve the over-fitting phenomenon. Unfortunately, these data enhancement methods are not practical in medical images. Because collecting medical images [such as computerized tomography (CT), tumor cells] is a time-consuming and laborious process, especially in the case of disease scarcity, patient privacy, and pathologist guidance. Therefore, increasing the sample size of medical images and improving the quality of medical images are essential for medical tasks. Zhao *et al.* (2019a) learned labeled example feature to synthesize unlabeled examples by building a model of transformations to increase the number of samples. Wolterink *et al.* (2017) used low-dose CT and routine-dose CT as data input to train an adversarial discriminator through a GAN network to evaluate routine-dose CT and reduce the noise of low-dose CT. In general, the GAN network relies on a large number of datasets and only can be applied to low-resolution images. To solve this problem, Baur *et al.* (2018) synthesized high-resolution skin pathology images with DDGAN under a small skin disease training sample.

Although their data enhancement methods have achieved remarkable results in medical images, they are still unsuitable for cluster cell data. There are two main reasons. First, they need a certain amount of data for labeling cluster cells, which inevitably faces the problem of difficulty in labeling cluster cells. Second, these data enhancement methods rely on network training, which will lead to the failure of synthetic data due to the instability and poor generalization of the network. When compared with these methods, we can synthesize cluster cells only with a small number of discrete labeled cells. The synthesized cluster cells have rich information of overlap, adhesion and noise, which can effectively fit the real data and alleviate the over-fitting phenomenon. In addition, the process is simple and effective without adding additional network training.

#### 1.1.2 Cluster cell overlapping occlusion segmentation

Overlapping occlusion phenomena are widespread. To deal with the high overlap phenomenon, Ke *et al.* (2021) and Lazarow *et al.* (2020) constructed the occluder and the occluded module based on the occlusion relationship. This module makes full use of interactive information through the relationship between two instances and achieved high-performance results on coco and cityscapes panoptical segmentation. In contrast, these phenomena also exist in medical images. Some scholars apply prior knowledge to cluster cell overlapping occlusion segmentation. Kong *et al.* (2011) calculated the segmentation boundary according to the concave point distance between overlapping instances. Song *et al.* (2018) designed an energy function for the fragment information of cluster cells, which can provide geometric information in overlapping contour segmentation. Although these methods have made some progress in cervical cancer cluster cell segmentation by using boundary, shape and geometric information, they perform poorly in complex situations. The key to this problem is that the network must have robust feature extraction ability, not a single information expression. In this problem, deep learning shows the powerful performance. Paulauskaite-Taraseviciene *et al.* (2019) used two different conceptual models of Unet and Mask region-based convolutional neural network (R-CNN) to jointly segment overlapping cluster cells and analyzed the performance of the deep learning method in this regard. Yi *et al.* (2019a) and Zhou *et al.* (2019b) used a two-stage instance segmentation network to extract the bounding boxes of multi-instance cells of the same, and performs contour segmentation for instance cells.

Different from the above methods, our anchor free KD can fully express intensity (or color) information and shape heterogeneity in the overlapping region, which makes our framework has strong robustness to complex textures and highly overlapping cells without any prior shape information.

#### 1.1.3 Cluster cells densely adhered segmentation

In the task of fine cell segmentation, an ongoing challenge is to segment densely contacted squeezed deformed cells and outline blurred cell boundaries. To solve this problem, Liu *et al.* (2019) added a dense connected conditional random field on a lightweight network to improve the segmentation precision. Graham *et al.* (2019) encoded the distance from the nucleus pixels to the centroid, and the distance information can assist in the accurate segmentation of overlapping instances. Chen *et al.* (2020b) designed a two-stage fine-grained segmentation network from coarse to fine: in the first stage, a network similar to the contour-aware informative aggregation mask net Zhou *et al.* (2019a) was used to obtain each instance in the cluster cells. In the second stage, the instance cells were refined through up-down sampling and residuals. Similarly, Fan *et al.* (2020) constructed an attention map to learn each instance end-to-end, it can effectively suppress the background and improve the recognition ability of overlapping instances.

However, it is worth mentioning that the above algorithms only are designed for feature extraction of fine information, which easily leads to over- or under-segmentation in case of low overlapping boundary gradient and heterogeneous cluster cell shape. So, we propose a CAC module to constrain the cell boundaries, which can improve the refine the ability to overlap adhesion boundaries to prevent over-segmentation or under-segmentation.

## 2 Methodology

**Fig. 2.**
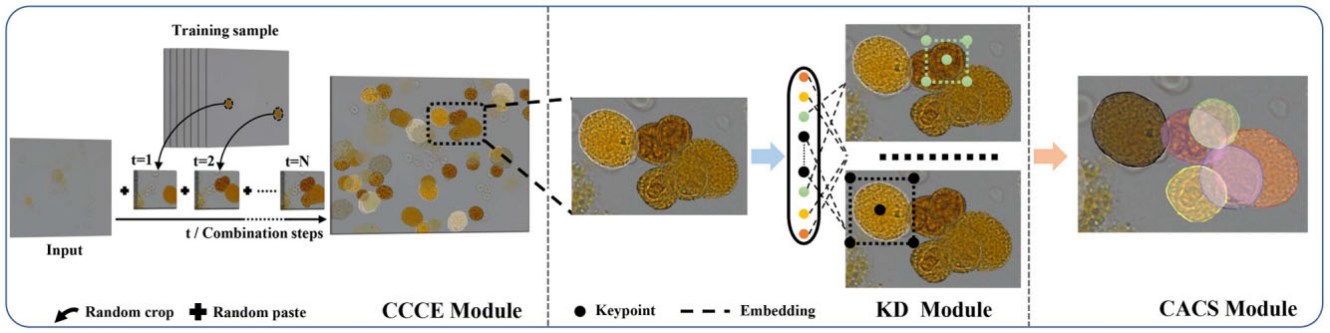
The overall flow chart of CC Framework. CC Framework comprises three stages: CCCE module enhance clusters attributes, KD module to obtain instance proposals and CACS module for instance cells segmentation

An overview of the CC Framework for cluster cells segmentation is shown in Figure 2. The framework comprises three parts: cluster cells combination enhance module (CCCE) to enhance clusters attributes as input data, the KD module implements the KD-based scheme to obtain instance proposals, following cluster cells segmentation by CACS module.

Algorithm 1: Procedure of CCCE

**Require:** training image $x_{0}$ and $x_{t}$ with *W* x *H*.

Let the $x_{t}$ contain K cells. Initial parameters *th*.

**Ensure:** Image of cell clusters $\tilde{x}$.

1: **for** *t* ← 1 to *N* **do**

2:  **for** *i* ← 0 to *K* **do**

3:   Rand_x=np.random.randint(0,W)

4:   Rand_y=np.random.randint(0,H)

5:   Crop_coord= $A(x_{k})$ ⊕ (Rand_x, Rand_y)

6:   Base_coord= $A(x_{0})$

7:   Overlap_coord= Crop_coord ∩ Base_coord

8:   $\tilde{x}$= $x_{0}$ ⊕ $x_{k}$  ⊙ Crop_coord

9:   $\tilde{x}$= $x_{0}$  ⊙ Overlap_coord/∂

10:   All_area=(Base_coord ∪ Crop_coord).sum()

11:   Overlap_coord= Crop_coord ∩ Base_coord

12:   **while** All_area ≤  *th* **do**

13:    **return**  $\tilde{x}$

14:   **end while**

15:  **end for**

16: **end for**

17: **return**  $\tilde{x}$

### 2.1 CCCE module

Different from cutout, cutmix and gridmask, CCCE is a simple and efficient data enhancement strategy. As shown in the left part of Figure 2, we select a very small number of data images from the pleural effusion tumor cell cluster dataset as training samples, which only contain a few cells. Specifically, we randomly select an initial image and N images from the training samples. Then gradually crop the area of the cell in N images, following paste the area of the cell randomly onto the initial image to generate the final cluster cell image. Let $x_{0}\in R^{W\times H\times C}$ and $x_{t}\in R^{W\times H\times C}$ denote the initial training image and the randomly selected training image. $\tilde{x}$ represents synthesized cells image by CCCE module, and the generation process of the CCCE module is illustrated in the Algorithm 1. Assuming that the $x_{t}$ contains K cell $x_{k}$, each cell $x_{k}$ can increase the sample diversity through various affine transformation methods such as random rotation, color jitter and Gaussian noise; *A* is the operation of obtaining cell coordinates. Base_coord, Crop_coord and Overlap_Coord represent the coordinates of $x_{0},\;x_{k}$ and overlapping regions, respectively. These coordinate points $\in {\left\{0,1\right\}}^{W\times H}$ denote a binary mask; ⊙, ⊕ is element-wise multiplication and addition. In our experiment, all training sample pixels are normalized to (0,1), which will lead to the pixel value of the overlapping area may be more than 1, and the visualization effect of this area does not conform to the real overlapping images, so we divide the overlapping area ∂ (1.8, 2.2, 2.5) to approximate the real images. Besides, the foreground area *th* will be set to control the continuation and termination of cell synthesis, which can effectively evade the imbalance of positive and negative samples.

### 2.2 KD module

**Fig. 3.**
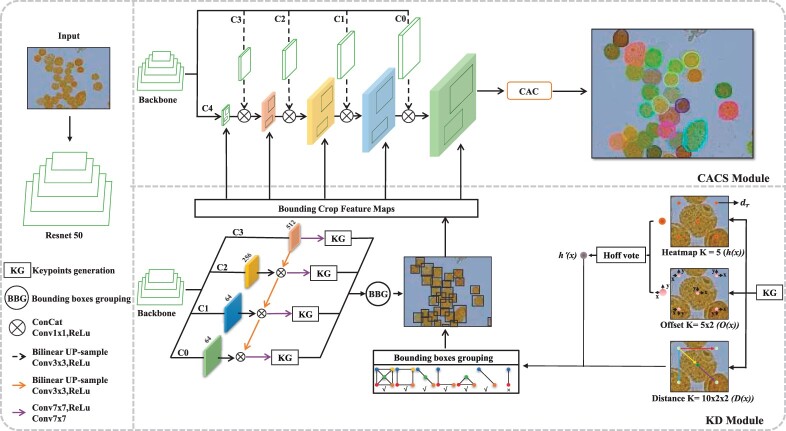
Overall network framework. The network consists of two modules: KD module, CACS module, they share a backbone Resnet 50

Our CC framework uses Resnet50 as the Backbone. To avoid missed and false detection, we detect five keypoints with embedding vector as the bounding boxes of cells by Keypoints generation (KG)—the top-left, top-right, bottom-left, bottom-right and the center points. These key points are grouped into cells bounding boxes using Bounding boxes grouping (BBG). The flowchart is shown in Figure 3 (KD module).

#### 2.2.1 Keypoints generation

Considering the multi-scale cells, we fuse the features of the backbone C0–C3 layer as to the input of KG. Heatmaps *h*(*x*), Offsets *O*(*x*) and Distance *D*(*x*) are outputs through conv7×7, relu and conv7×7. Heatmaps are used to commonly represent the position of key nodes in human posture estimation Newell *et al.* (2016). Similarly, in our KD, heatmap *h*(*x*) also represents the possible corners cells bounding boxes, which outputs five channels to represent keypoint categories. To create a heatmap, each channel contain disc $d_{r}y=\{x:\left\|x-y\right\|\leq r\}\}$, where *y* and *r* are the position of the keypoint and radius of the disc respectively, and *h*(*x*) = 1 for $x\in d_{r}(y)$, otherwise *h*(*x*) = 0. Besides, our KD involves downsampling and upsampling to merge different scale receptive field. When we map a location $\left\lfloor \frac{y}{n}\right\rfloor$ from the locations *y* in the heatmap *h*(*x*) (*n* refer to sampling factor), some precision may be lost. Hence, to improve the accuracy prediction, we predict Offsets *O*(*x*) with 5×2 channels to punish the heatmap *h*(*x*) locations deviation loss and set multi-radius on multi-scale features to supervise 2D positions of a keypoint y from coarse to fine.

(1) $$O(x)=\left(\frac{y}{n}-\left\lfloor \frac{y}{n}\right\rfloor \right),x\in d_{r}(y)$$

where *O*(*x*) encodes the displacement between unrounded coordinates $\frac{y}{n}$ and rounded coordinates $\left\lfloor \frac{y}{n}\right\rfloor$. We apply binary cross-entropy and L1 loss to punish heatmap *h*(*x*) and offset *O*(*x*) the loss. In the actual training process, multiple interference points will be generated due to the influence of noise. Therefore, we combine heatmaps *h*(*x*) and offsets *O*(*x*) to obtain the keypoint score map h′(x) by using Hough calculators Papandreou *et al.* (2018), then filter the interference points through the threshold.

(2) $$h\prime (x)=\frac{1}{\pi r^{2}}\sum_{k=1}^{N}h(x_{k})B(x_{k}+o(x_{k})-x)$$

here, $x_{k}$ represents the *k*th key point of the image, *B* denotes the bilinear interpolation kernel.

#### 2.2.2 Bounding boxes grouping

After obtaining the score keypoint h′(x), we sort the keypoint score map h′(x) in descending order and greedily connect the (*k*, *l*) pair of keypoints belonging to the same object by the prediction graph distance $D_{k,l}(x)$.

(3) $$D_{k,l}(x)=(y_{l}-x),x\in d_{r}(y_{k})$$

where *k* and *l* represent two key points, respectively. The direction of the connection keypoint is similar to a directed graph. Therefore, there are 10×2×2 channels (10×2 connection types, two direction in the *X*- and *Y*-axis). Similar to offset, we use the L1 to punish prediction graph distance $D_{k,l}(x)$ loss.

Various groups are obtained by the above connect methods, shown in Figure 3 (BBG). To reduce the possibility of losing box proposals, we select 5–2 diagonal keypoints as a box; then use Diou NMS to inhibit repeated detection for the same object.

### 2.3 CACS module

**Fig. 4.**
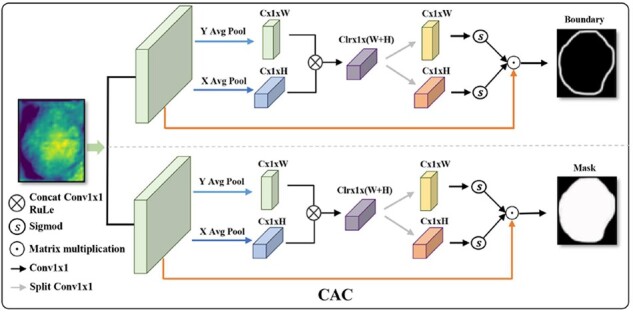
Contour attention constraint module (CAC)

For cluster cells segmentation, over- and under-segmentation problems often occur in the highly overlapping area. To overcome the drawbacks mentioned above, we propose a CACS module shown in Figure 3 (CACS module), which can be divided into the following steps: (i) Feature extraction. We crop and fuse the multi-scale features from the backbone by the KD detector. These fused features have deep semantic information and shallow boundary information. (ii) CAC. We propose the CAC module focus on the cell’s boundaries in Figure 4. The location information of fused feature $X\in R^{C\times H\times W}$ will be extracted by the CA attention Hou *et al.* (2021). Specifically, CA attention using Average Pooling along horizontal and vertical directions encode the fused feature *X* to capture location information $X_{x}\in R^{C\times 1\times W}$ and $X_{y}\in R^{C\times H\times 1}$. Because the cell features are represented by the neighborhood pixels in the local region, we fuse the location information $X_{x}$ and $X_{y}$ to enhance location feature correlation. Then, using 1×1 Conv and Sigmoid to obtain boundary attention weight. Finally, a multiplication of the attention weight and fused feature can help the network improve the ability to identify overlapping adhesion areas. Besides, the output of the CAC module is cells boundary and cells mask. First, the boundary constraint can further punish the loss of over- and under-segmentation. Second, the influence of boundary constraint is not only reflected in the segmented network but also can further improve the perception keypoints in the process of updating shared network parameters.

## 3 Experiment

In this section, extensive experiments have been conducted to demonstrate the effectiveness of the CC framework. We first briefly introduce the experiments datasets, followed by evaluating metrics. Then, we show the implementation and training details. In addition, we provide a summary of the evaluation for our framework. Ablation studies that aim to demonstrate the effectiveness of each component in CC framework are also provided.

### 3.1 Datasets

**PETCCD.** We constructed a pleural effusion tumor cluster cell dataset (PETCCD). This dataset is divided into two types: discrete pleural effusion tumor cells (DPETC, 68 training samples) and aggregated pleural effusion tumor cluster (APETC, 109 images). To verify the effectiveness of our proposed the CCCE module, we define the synthetic data through the CCCE module as combined enhancement of pleural effusion tumor cluster (CEOPETC, 200 training samples). For these datasets, we all use the APETC dataset as a validation dataset (24 images) with this number of samples selected by 109 images and remaining 85 samples for training.

**OCWBC.** Overlapping cluster of white blood cell (OCWBC) dataset is a public dataset and can be used for cell instance segmentation. It includes two categories: discrete white blood cells (DWBC, 25 training samples), dense OCWBC (DOCWBC, 78 images). we synthesized 250 training samples on DWBC, combined enhancement of OCWBC, (CEOOCWBC). Similar to PETCCD, this validation dataset (24 images) is selected by DOCWBC, remaining 54 samples for training.

### 3.2 Experiment configuration

Our experiments are dependent on Python 3.6.0, CUDA10.0, Cudnn7.6.5 and Pytorch1.6.0. The experimental equipment is 12G Titan V GPU, Intel Core i9-7900X CPU and Unbuntu 18.04.

In the training process, we resize the equal aspect ratio of the input network image to 512×512. In addition, data enhancement methods, such as random expanding, clipping, flipping, contrast distortion, are used to increase the sample diversity and mitigate model overfitting. GT boxes are used as bounding boxes to train the CACS module. Further, we set a total of 200 epochs. The first 100 epochs module freeze boundary prediction and the last 100 epochs unfreeze boundary prediction. In the testing process, the input image is detected to obtain bounding boxes, which are then mapped to the detected bounding boxes to the CACS module for instance segmentation cells. lr is set to 0.0001. The batch size is set to 4. Diou NMS and segmentation threshold is set to 0.5.

### 3.3 Boundary segmentation evaluation metrics

In the instance segmentation task, most papers take *AP*^mask^ as the metrics to evaluate their algorithms. *AP*^mask^ calculates precision and recall curve through Mask IoU (intersection-over-Union). When compared with the pixel level IoU of semantic segmentation, the response of Mask IoU to the boundary quality of objects with different scales is uneven. The segmentation quality of prediction boundary pixels cannot be accurately evaluated. Based on the above analysis, Boundary IoU (Cheng *et al.*, 2021) is proposed, which is sensitive to objects of multiple scales and will not punish anyone excessively. Mask IoU and boundary IoU are calculated as follows (4 and 5).

(4) $$M\mathrm{ask}\;IoU(G,P)=\frac{\left|G_{m}\cap P_{m}\right|}{\left|G_{m}\cup P_{m}\right|}$$

 

(5) $$B\mathrm{oundary}\;IoU(G,P)=\frac{\left|(G_{d}\cap G_{m})\cap (P_{d}\cap P_{m})\right|}{\left|(G_{d}\cap G_{m})\cup (P_{d}\cap P_{m})\right|}$$

where $G_{m}$ and $P_{m}$ respectively refer to the gt and predicted mask, $G_{d}$ and $P_{d}$ are the sets of all pixels within d pixels distance from the $G_{m}$ and $P_{m}$ contours respectively. We choose *AP*^mask^ and $AP_{\text{TP}}^{\text{boundary}}$ as segmentation quantitative evaluation criteria. *AP*^mask^ is the standard evaluation metrics for instance segmentation (Lin *et al.*, 2014). $AP_{\text{TP}}^{\text{boundary}}$ is employed to calculate the positive sample (averaged over Boundary IoU thresholds), which can eliminate the influence of negative sample detection object on boundary segmentation evaluation to improve the segmentation evaluation standard.

### 3.4 Experimental results

**Fig. 5.**
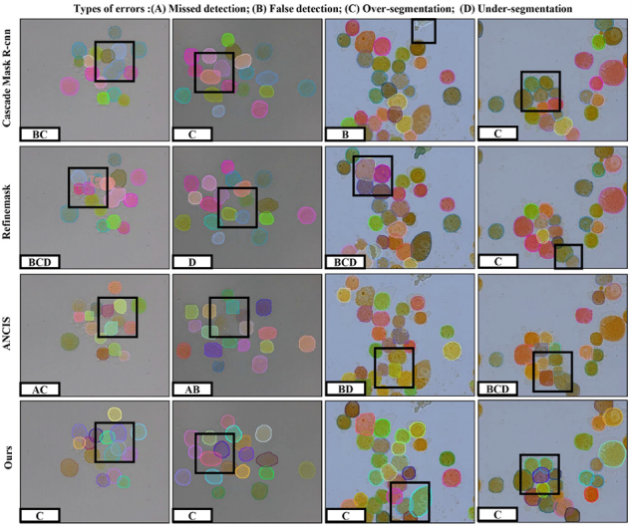
Instance segmentation qualitative results for cluster cells on PETCCD dataset

**Fig. 6:**
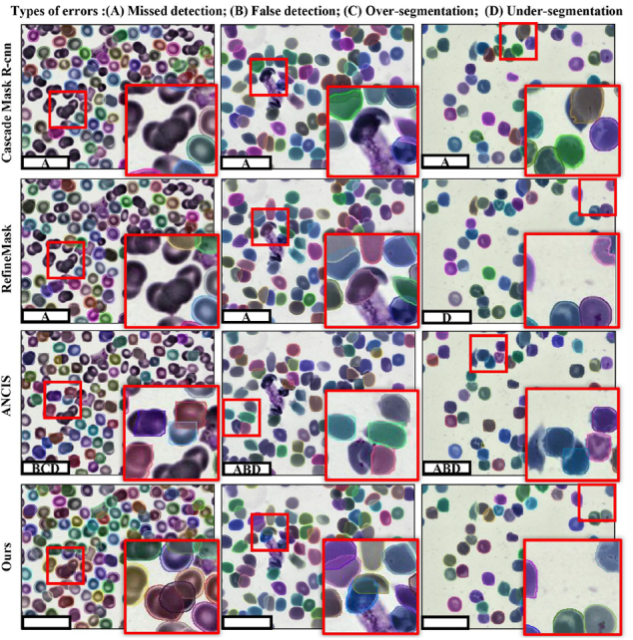
Instance segmentation qualitative results for cluster cells on OCWBC dataset

We compare the proposed CC framework with state-of-art algorithms on CEOPETC and CEOOCWBC datasets. It can be observed from Table 1 that our method has achieved the more than 90% results on *AP*^mask^, much better than other algorithms. For the comparison of *AP*^boundary^, although only by calculating positive samples, our method still is a reduction of 2.74–1.0% on the highest RefineMask, and the overall results still outperform other instance segmentation algorithms. Besides, to visually compare our method with some competitive algorithms, partial better segmentation qualitative results are shown in Figures 5 and 6. For the PETCCD and the OCWBC datasets, most comparison algorithms suffer from huge multi-instance object confusion recognition (False detection) and loss of prediction target (Missed detection), especially on overlap and adhesion region. Similarly, these algorithms are unable to differentiate obscure cell boundaries, which may lead to over- and under-segmentation for clusters cell pixel. On the contrary, compared with these SOTA methods, our framework exhibit remarkable performance in capturing tiny and fuzzy boundary information of clusters cells, barely false detection, missed detection and over-segmentation.

**Table 1.** Performance comparison of the proposed framework and the state-of-the-art methods on CEOPETC and CEOOCWBC dataset. *AP^m^* refer to *AP*^mask^

Networks	Datasets	0.5*AP^m^*	0.7*AP^m^*	0.5$AP_{TP}^{b}$	0.7$AP_{TP}^{b}$
Yolact Bolya *et al.* (2019)	**CEOPETC**	78.90	54.63	80.99	83.47
Cascade Mask R-CNN Cai and Vasconcelos (2021)		84.53	83.43	86.85	87.33
SOLOv2 Wang *et al.* (2020)		61.20	43.80	56.60	34.60
RefineMask Zhang *et al.* (2021)		81.51	80.05	**87.47**	**88.05**
ANCIS Cheng and Qu (2020)		80.97	79.90	81.33	81.33
**Ours**		**97.52**	**96.52**	86.40	87.05
Yolact Bolya *et al.* (2019)	**CEOOCWBC**	70.76	84.79	53.55	87.85
Cascade Mask R-CNN Cai and Vasconcelos (2021)		88.40	78.00	89.67	90.24
SOLOv2 Wang *et al.* (2020)		83.90	58.20	74.40	39.10
RefineMask Zhang *et al.* (2021)		79.46	77.70	**90.84**	**91.83**
ANCIS Cheng and Qu (2020)		71.79	67.00	81.23	81.23
**Ours**		**93.65**	**91.45**	89.77	89.09

*N* *ote*: $AP_{TP}^{b}$ refer to $AP_{\text{TP}}^{\text{boudnary}}$

In addition, to evaluate the effectiveness of the CCCE module, We compare the results on the synthesized datasets (CEOPETC, CEOPETC) with those of original datasets, as shown in Table 2. It can be immediately noticed that the segmentation results of synthesized datasets outperform discretely cell datasets (DPETC, DWBC), even higher than the aggregate cell dataset (APETC, CEOOCWBC) in some metrics. The experimental results demonstrate the significant advantages of the CCCE module in segmenting the cluster cells with scarce data.

**Table 2.** Performance comparison of the proposed CCCE module on the PETCCD and OCWBC datasets by state-of-the-art methods

Networks	Datasets	0.5*AP^m^*	0.7*AP^m^*	0.5$AP_{TP}^{b}$	0.7$AP_{TP}^{b}$	Datasets	0.5*AP^m^*	0.7*AP^m^*	0.5$AP_{TP}^{b}$	0.7$AP_{TP}^{b}$
Yolact (Bolya *etal.*, 2019)	DPETC	61.14	46.60	77.89	80.84	DWBC	70.25	39.81	79.54	84.51
	**CEOPETC**	78.09	54.63	80.99	83.47	**DOCWBC**	**70.76**	**84.79**	53.55	**87.85**
	APETC	**83.69**	**55.98**	**81.10**	**83.71**	CEOOCWBC	70.39	41.57	**81.16**	86.31
Cascade Mask R-CNN (Cai and Vasconcelos, 2021)	DPETC	67.98	48.33	79.51	63.40	DWBC	77.89	63.98	86.30	87.96
	**CEOPETC**	84.53	**83.42**	86.85	87.33	**DOCWBC**	**88.40**	**78.00**	**89.67**	**90.24**
	APETC	**84.81**	82.82	**88.72**	**89.13**	CEOOCWBC	88.19	76.65	88.45	89.43
SOLOv2 (Wang *et al.*, 2020)	DPETC	11.50	02.69	08.20	01.30	DWBC	50.70	18.70	50.60	18.50
	**CEOPETC**	**61.20**	**43.80**	**56.60**	**34.60**	**DOCWBC**	**83.90**	**58.20**	**74.40**	**39.10**
	APETC	52.00	29.00	45.90	26.90	CEOOCWBC	83.90	58.20	74.40	39.10
Refine Mask (Zhang *et al.*, 2021)	DPETC	43.87	31.67	76.72	31.45	DWBC	70.32	59.38	88.03	89.33
	**CEOPETC**	**81.51**	80.50	**87.47**	88.05	**DOCWBC**	**79.46**	**77.70**	**90.84**	**91.83**
	APETC	71.03	**90.16**	70.85	**90.56**	CEOOCWBC	75.19	72.13	90.34	91.45
ANCIS (Cheng and Qu, 2020)	DPETC	78.12	78.71	80.11	80.11	DWBC	71.79	59.32	80.60	80.60
	**CEOPETC**	**80.07**	**79.90**	81.33	81.33	**DOCWBC**	**71.79**	67.00	81.23	81.23
	APETC	80.10	79.84	**82.87**	**82.87**	CEOOCWBC	71.19	**67.53**	**81.39**	**81.39**
**Ours**	DPETC	83.28	82.93	84.43	84.56	DWBC	89.37	84.58	88.84	89.53
	**CEOPETC**	**97.52**	**96.52**	86.40	87.05	**DOCWBC**	**93.65**	**91.45**	**89.77**	**90.18**
	APETC	96.22	94.32	**92.05**	**90.80**	CEOOCWBC	92.80	90.30	89.76	90.05

### 3.5 Ablation experiment

In this section, to justify the effectiveness of the CAC module, we performed ablation experiments on PETCCD and OCWBC datasets. Table 3 shows the results of the ablation experiment. We can notice that after adding the CAC module to the baseline, the results of PETCCD and CEOOCWB both increase by 0.74–3.38% on the *AP*^mask^. Simultaneously, the results on $AP_{\text{TP}}^{\text{boundary}}$ also increased by 0.01–0.79%. Therefore, this demonstrates that the proposed CAC module can further restrict the segmentation boundary and improve the expression ability of keypoint features.

**Table 3.** Ablation study for CAC module on CEOPET and CEOPET datasets

Datasets	CAC	0.5*AP^m^*	0.7*AP^m^*	0.5$AP_{TP}^{b}$	0.7$AP_{TP}^{b}$
CEOPET	×	95.89	93.14	85.97	86.55
	√	97.52 **(+1.63)**	96.52 **(+3.38)**	86.40 **(+0.79)**	87.05 **(+0.5)**
CEOOCWB	×	92.91	89.45	89.76	90.16
	√	93.65 **(+0.74)**	91.45 **(+2.00)**	89.77 **(+0.01)**	90.18 **(+0.02)**

### 3.6 Discussion

As can be seen from Table 1 and Figures 5 and 6, the anchor methods, such as Cascade Mask R-CNN, RefineMask etc, tends to missed and false detection on the overlap and adhesion regions. One possible reason would be that this method is difficult to select the appropriate initial anchor size and quantity to match each cell in the area of the intensive cells. Therefore, the offset of predicted bounding boxes will be caused by regression anchor. At the same time, in the keypoint detection algorithm, the accuracy of AP is directly related to the location and the connection mode of the keypoints. For ANCIS, keypoints position deviate gt boxes, so the bounding boxes connected by these key points cannot accurately cover each cluster cell. Compare these methods, our KD-based multi-scale feature fusion and boundary constraints can improve the expression ability of the keypoints feature. Following two to five keypoints combination and DIoU NMS, the detector can effectively filter redundant bounding boxes, retaining high score predicted bounding boxes. The quantitative results show that our detector can effectively avoid missed and false detection. In addition, the boundary of cluster cells in out-of-focus images tends to be blurry, relying on regressing mask is not enough to extract the detailed features of fuzzy boundary. Therefore, these comparison algorithms tend to be over- and under-segmentation. Thus we introduce the CAC module that can encode the detailed boundary feature and punish the boundary loss. From Table 3 and Figures 5 and 6, we can observe that our methods with the CAC module perform better than SOTA algorithms.

## 4 Conclusion

Accurately segmenting the cluster cells can help clinicians pay more attention to the lesion area and achieve a more accurate diagnosis of lung cancer. In this article, we propose a novel CC framework, which leverages the cluster cell combination enhance for more efficient miscellaneous cells feature representation. Quantitative and qualitative results demonstrate the advantages of our method in segmenting the instances cluster cells. In the future, we plan to construct a multi-category, sample imbalanced cluster cell dataset, which is more consistent with clinical medical pathological images. In addition, we will pay more attention to semi-supervised method to reduce the complexity of labeling data as well as improving model ability to adaptively learn cluster properties features.

## Funding

This work was supported by the National Natural Science Foundation of China [Grant Nos. 62020106004, 92048301 and 61906133].

*Conflict of Interest*: The authors declare that they have no known competing financial interests or personal relationships that could have appeared to influence the work reported in this paper.

## Data availability

The data underlying this article will be shared on reasonable request to the corresponding author.
